# Synthesis, *in Vitro* Antimycobacterial and Antibacterial Evaluation of IMB-070593 Derivatives Containing a Substituted Benzyloxime Moiety

**DOI:** 10.3390/molecules18043872

**Published:** 2013-03-28

**Authors:** Zengquan Wei, Jian Wang, Mingliang Liu, Sujie Li, Lanying Sun, Huiyuan Guo, Bin Wang, Yu Lu

**Affiliations:** 1 Institute of Medicinal Biotechnology, Chinese Academy of Medical Sciences and Peking Union Medical College, Beijing 100050, China; 2 The No.5 Hospital of Harbin, Harbin 150040, China; 3 Beijing Key Laboratory of Drug Resistance Tuberculosis Research, Department of Pharmacology, Beijing Tuberculosis and Thoracic Tumor Research Institute, Beijing Chest Hospital, Capital Medical University, Beijing 101149, China

**Keywords:** IMB-070593 derivatives, benzyloxime, synthesis, antimycobacterial activity, antibacterial activity

## Abstract

A series of novel IMB-070593 derivatives containing a substituted benzyloxime moiety and displaying a remarkable improvement in lipophilicity were synthesized and evaluated for their *in vitro* antimycobacterial and antibacterial activity. Our results reveal that the target compounds **19a**–**m** have considerable Gram-positive activity (MIC: <0.008–32 µg/mL), although they are generally less active than the reference drugs against the Gram-negative strains. In particular, compounds **19h**, **19j**, **19k** and **19m** show good activity (MICs: <0.008–4 µg/mL) against all of the tested Gram-positive strains, including ciprofloxacin (CPFX)- and/or levofloxacin (LVFX)-resistant MSSA, MRSA and MSSE. Moreover, compound **19l** (MIC: 0.125 µg/mL) is found to be 2–4 fold more active than the parent IMB070593, CPFX and LVFX against *M. tuberculosis* H37Rv ATCC 27294.

## 1. Introduction

As one of the largest classes of antimicrobial agents quinolones have been known for 50 years. These antibiotics, which inhibit the type II bacterial topoisomerases DNA gyrase and topoisomerase IV, are used mainly to fight both community-acquired and serious hospital-acquired infections [1]. On the other hand, DNA gyrase is considered to be the sole topoisomerase drug target of fluoroquinolones in *Mycobacterium tuberculosis* (MTB) [2]. Ciprofloxacin (CPFX), ofloxacin and sparfloxacin were recommended by the World Health Organization in 1996 as second-line agents for the treatment of tuberculosis (TB), mainly in cases involving resistance or intolerance to first-line anti-TB therapy [3]. Moreover, two newer C-8 methoxyfluoroquinolones, moxifloxacin (MXFX) and gatifloxacin, possessing a particularly strong *in vitro* and *in vivo* activity against MTB [4,5] are currently being further evaluated as anti-TB drugs.

However, since the mid-1990s, quinolone resistance started to increase in almost all Gram-positive and Gram-negative species as well as MTB [6,7]. The continued increase in resistance has put enormous pressure on public health systems worldwide [8], predominantly due to the high level of use and to some degree of abuse. Although considerable results have been achieved recently, there is an urgent need for the discovery and development of effective novel fluoroquinolones to confer desirable biological and pharmacological properties. Clearly, a more practical strategy is to modify the structures of existing fluoroquinolones to increase potency and overcome resistance.

Recently, a great number of syntheses of fluoroquinolone derivatives have been reported, together with the corresponding structure-activity relationship (SAR) studies [9,10,11]. As a result of these SAR studies, it appears evident that the substituent at C-7 position, the only area that substitution of bulky functional group is permitted, plays an important role in the antibacterial potency, antibacterial spectrum and toxicity of fluoroquinolones [12]. Moreover, it is generally believed that simply increasing the lipophilicity could also improve the anti-MTB and antibacterial activity of fluoroquinolones by introduction of an additional functional moiety on the primary or second amino group of the C-7 side chain [13,14,15,16,17,18]. Therefore, reasonable modification at C-7 position is likely to produce more effective anti-TB and antibacterial agents.

In our previous paper, we reported a series of gemifloxacin (GMFX) derivatives with remarkable improvement in lipophilicity and several target compounds featuring a substituted benzyloxime-incorporated pyrrolidino-substitution at C-7 position have superior Gram-positive activity to the corresponding methyloxime analog (GMFX) [19]. Similarly, some fluoroquinolone derivatives bearing a 3-(substituted benzyloximido)-2-(aminomethyl)azetidin-1-yl group at the C-7 position were found to be far more active than the corresponding methyloxime analog against Gram-positive strains in our study [1].

**Figure 1:**
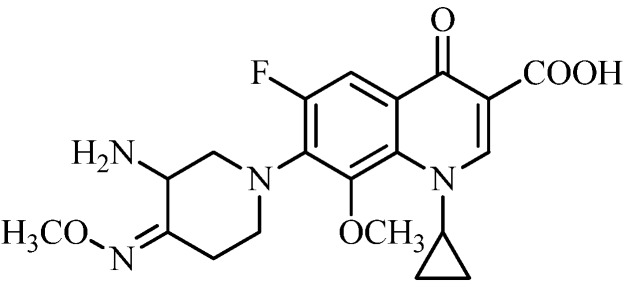
Structure of IMB-070593.

Inspired by the above research results with azetidinyl- and pyrrolidinyl-based fluoroquinolones, we planned to make structural modifications on IMB-070593 (Figure 1), a piperidinyl-based fluoroquinolone candidate discovered in our lab. In late pre-clinical stage of development currently, IMB-070593 possesses potent *in vitro* and *in vivo* antibacterial activity [20] and *in vitro* anti-MTB activity [21] as well as extremely low phototoxicity, hepatotoxicity and cardiac toxicity (unpublished data). Given that replacement of methyloxime of IMB-070593 by aliphatic moieties such as ethyl group with slightly increased lipophilicity, has almost no impact on the antibacterial activity [20], a series of novel IMB-070593 derivatives were designed, synthesized by introduction of diversified more lipophilic benzyloximes instead of methyloxime of the piperidine ring (Figure 1) in this study. Our primary object was to optimize the potency of IMB-070593 against MTB and clinically important pathogens including methicillin-resistant *S. aureus* (MRSA).

## 2. Results and Discussion

### 2.1. Chemistry

**Scheme 1:**
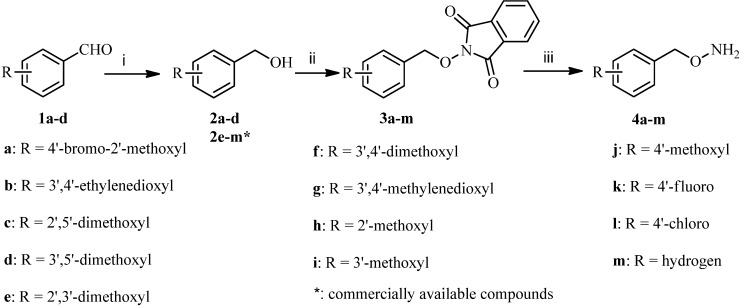
Synthesis of *O*-benzylhydroxylamines **4a**–**m**.

Commercially unavailable *O*-benzylhydroxylamines **4a**–**m** were first prepared according to Scheme 1. Reduction of various benzaldehydes **1a**–**d** with sodium borohydride in methanol gave the phenylmethanols **2a**–**d**, and then **2a**–**d** and commercially available compounds **2e**–**m** were coupled with 2-hydroxyisoindoline-1,3-dione in the presence of diethylazodicarboxylate (DEAD) and triphenylphosphine (PPh_3_) in tetrahydrofuran to produce condensates **3a**–**m**. The desired compounds **4a**–**m** were obtained by treatment of **3a**–**m** with hydrazine hydrate in dichloromethane according to well established procedures [22].

*Reagents and conditions*: (i) NaBH_4_, CH_3_OH, rt, 2 h, 84%–90%; (ii) 2-hydroxyisoindoline-1,3-dione, PPh_3_, DEAD, THF, 0 °C, 1 h, 79%–85%; (iii) N_2_H_4_^.^H_2_O, CH_2_Cl_2_, rt, 2 h, 89%–92%.

**Scheme 2:**
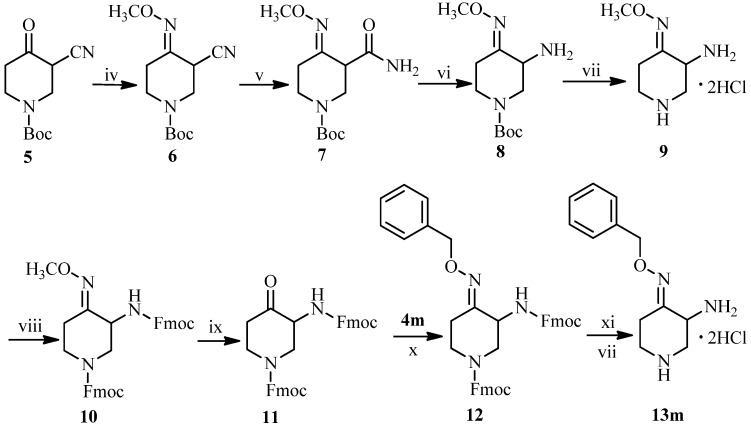
Synthesis of piperidine derivative **13m**.

**Scheme 3:**
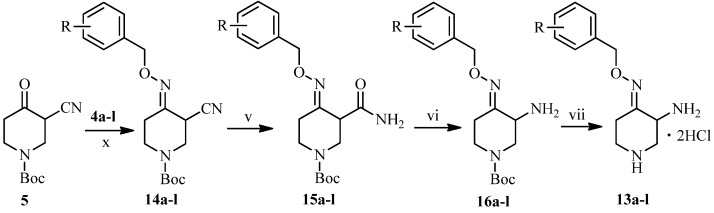
Synthesis of piperidine derivatives **13a**–**l**.

**Scheme 4:**
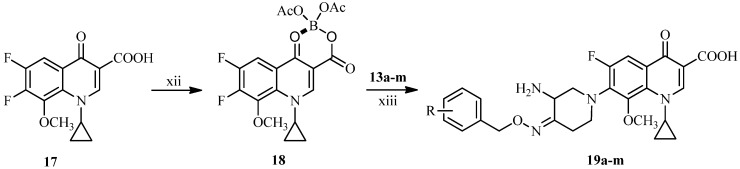
Synthesis of target compounds **19a**–**m**.

Detailed synthetic pathways to novel 3-amino-4-benzyloxyimino-piperidines **13a**–**m** and IMB-070593 derivatives **19a**–**m** are depicted in Scheme 2, Scheme 3, Scheme 4, respectively. The synthesis of **13m** is illustrated in Scheme 2. We previously reported a low total yield (<5%) synthetic route to the key intermediate **9** from ethyl 1-benzyl-4-oxopiperidine-3-carboxylate via a 9-step procedure [20]. In order to overcome its disadvantages, a simpler route for synthesis of **9** was developed in this work. Oximation of readily available *tert-*butyl 3-cyano-4-oxopiperidine-1-carboxylate (**5**) [23] followed by treatment with DMSO-H_2_O_2_-K_2_CO_3_ system gave amide **7**. Hoffmann degradation of the amide **7** was conducted successfully using freshly prepared sodium hypobromite instead of sodium hypochlorite to yield primary amine **8**. The *N*-*tert*-butoxycarbonyl (Boc) protecting group on amine **8** was removed with hydrogen chloride gas in methylene chloride to afford the side chain compound **9** of IMB-070593 in good total yield (>20%, from **5**).

*Reagents and conditions*: (iv) CH_3_ONH_2_, HCl, NaOH, CH_3_OH, 50 °C, 3 h, 93%; (v) H_2_O_2_, K_2_CO_3_, DMSO, rt, 2 h, 71%; (vi) NaBrO, CH_3_CN, rt, overnight, 63%; (vii) HCl gas, CH_2_Cl_2_, rt, 3h, 52%; (viii) FmocCl, Et_3_N, CH_2_Cl_2_, rt, 57%; (ix) Ethyl acetoacetate, 36% HCl, CH_3_OH, 60 °C, 4 h, 52%; (x) C_2_H_5_OH, rt, 5 h, 75%; (xi) 10%NaOH, THF, reflux, 10 h, 45%.

However, the conversion of the methyl oxime **8** or **9** into the corresponding ketone by acidic hydrolysis turned out to be rather complicated and the products difficult to purify. Even though various acids (HCl, HBr, HI, H_2_SO_4_, CH_3_SO_3_H) in different solvents (H_2_O, CH_3_OH, C_2_H_5_OH) were used for the hydrolysis, we were not able to obtain the desired product in acceptable yield. Therefore, the primary and second amino groups of **9** had to be protected by treatment with 9-fluorenylmethyl chloroformate (FmocCl) to form the bis-Fmoc protected methyloxime **10**, which upon hydrolysis afforded the desired ketone **11** successfully albeit in poor yield (<30% for the two steps). Oximation of the ketone **11** with *O*-benzylhydroxylamine **4m** in ethanol followed by deprotection of the bis-Fmoc groups on the amine **12** in NaOH-THF system provided 3-amino-4-(benzyloxyimino)piperidine dihydrochloride (**13m**).

The synthesis of the other side chain compounds **13a**–**l** is illustrated in Scheme 3. Reaction of ketone **5** with various substitueted *O*-benzylhydroxylamines **4a**–**l** followed by treatment with DMSO-H_2_O_2_-K_2_CO_3_ system afforded amides **15a**–**l**. Hoffmann degradation of **15a**–****l and subsequently deprotection of the resulting amines **16a**–**l** gave 3-amino-4-(substituted benzyloxyimino)piperidine dihydrochlorides **13a**–**l**.

*Reagents and conditions*: (x) C_2_H_5_OH, rt, 5 h, 90%–95%; (v) H_2_O_2_, K_2_CO_3_, DMSO, rt, 2 h, 71%–75%; (vi) NaBrO, CH_3_CN, rt, overnight, 59%–65%; (vii) HCl gas, CH_2_Cl_2_, rt, 3 h, 50%–56%. See Scheme 1 for structures of the groups **a**–**l**.

Finally, as illustrated in Scheme 4, the novel IMB-070593 derivatives **19a**–**m** were obtained by coupling the side chain compounds **13a**–**m** with the boric chelate **18** of the fluoroquinolone nucleus **17** according to well-established literature procedures [12,24].

*Reagents and conditions*: (xii) H_3_BO_3_, (CH_3_CO)_2_O, CH_3_CO_2_H, 100 °C, 2 h, 70%; (xiii) (1) Et_3_N, CH_3_CN, 50 °C, overnight; (2) 5% NaOH, 50 °C, 1 h, 18%–34% (for two steps). See Table 1 for structures.

Because the oxime group is present in the *E-* or *Z-* configuration, it was necessary to determine the geometries of all the oxime target compounds **19a**–**m**. Although we were unable to prepare X-ray-quality single crystals of any oxime intermediate or product in this study, we had previously obtained single crystals of 4-(methoxyimino)-3-methylaminopiperidinone dihydrochloride, an *N*-methylated **9** derivative, in which the piperidine ring adopts a chair conformation and the methyloxime geometry exists in an *E*-configuration [20]. Accordingly, we can speculate that the oxime group of the target compounds in this study should have the same *E*-configuration due to single signals of the piperidine ring observed in the ^1^H-NMR spectra of the compounds.

### 2.2. Lipophilicity

Lipophilicity of the target compounds **19a**–**m** and the parent IMB-070593 is expressed in the term of their ClogP values which were calculated by Chemoffice 2010 software. As shown in Table 1, there is a remarkable improvement in the lipophilicity of the derivatives **19a**–**m** as evidenced by their ClogP values (0.60–1.86) which are much more than that of IMB-070593 (−0.72) (statistically significant at *p* < 0.001 using t-test) (Table 1).

**Table 1:** Structures, lipophilicity and antimycobacterial activity of compounds **19a**–**m**. 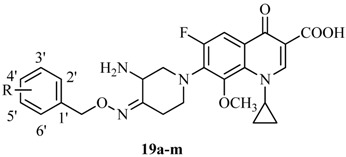

Compd.	R	Clog p ^a^	MIC (µg/mL)	
			MTB ^b^	MDR-MTB ^c^
**19a**	4′-bromo-2′-methoxyl	1.86	16	16
**19b**	3′,4′-ethylenedioxyl	0.87	8	4
**19c**	2′,5′-dimethoxyl	0.95	8	8
**19d**	3′,5′-dimethoxyl	0.95	16	4
**19e**	2′,3′-dimethoxyl	0.60	8	8
**19f**	3′,4′-dimethoxyl	0.60	8	8
**19g**	3′,4′-methylenedioxyl	0.91	4	1
**19h**	2′-methoxyl	0.86	8	4
**19i**	3′-methoxyl	0.86	4	1
**19j**	4′-methoxyl	0.86	4	2
**19k**	4′-fluoro	1.08	4	4
**19l**	4′-chloro	1.65	0.125	4
**19m**	hydrogen	0.94	4	2
**IMB**		−0.72	0.25	0.125
**CPFX**			0.5	0.25
**LVFX**			0.25	0.125
**MXFX**			0.125	0.125
**INH**			0.06	2
**RIP**			0.06	>40

IMB, IMB070593; CPFX, ciprofloxacin; LVFX, levofloxacin; MXFX, moxifloxacin; RIP, rifampicin; INH, isoniazid; ^a^ The Clog p is calculated by Chemoffice 2010 software; ^b^ MTB: MTB H37Rv ATCC 27294; ^c^ MDR-MTB: MDR-MTB 20161 is resistant to RIP and INH.

### 2.3. Anti-MTB Activity

The target compounds **19a**–**m** were initially evaluated for their *in vitro* activity against MTB H37Rv ATCC 27294 and MDR-MTB 20161 clinical isolate using the Microplate Alamar Blue Assay (MABA) [25,26]. The minimum inhibitory concentration (MIC) is defined as the lowest concentration effecting a reduction in fluorescence of ≥90% relative to the mean of replicate bacterium-only controls and MICs of the compounds **19a**–**m** along with CPFX, levofloxacin (LVFX), MXFX, isoniazid (INH) and rifampicin (RIP) for comparison are presented in Table 1.

The data reveal that the target compounds **19a**–**m** have considerable activity against both of the tested MTB strains (MICs: 0.125–16 µg/mL). As for MTB H37Rv ATCC 27294, the most active compound **19l** (MIC: 0.125 µg/mL) was found to be 2–4 fold more potent than the parent IMB070593, CPFX and LVFX (MICs: 0.25–0.5 µg/mL), and comparable to MXFX (MIC: 0.125 µg/mL), the fluoroquinolone with the strongest anti-MTB activity. 

In the case of MDR-MTB 20161 clinical isolate resistant to RIP and INH, the target compounds **19a**–**m** (MICs: 1–16 µg/mL) show less active than the parent IMB070593 and the three other reference fluoroquinolones, but compounds **19g** and **19i** have useful activity (MICs: 1 µg/mL) against this strain.

### 2.4. Antibacterial Activity

The target compounds **19a**–**m** were evaluated for their *in vitro* antibacterial activity against representative strains using standard techniques [27]. Minimum inhibitory concentration (MIC) is defined as the concentration of the compound required to give complete inhibition of bacterial growth, and MIC values of **19a**–**m** against Gram-positive and Gram-negative strains along with the parent IMB-070593, CPFX and LVFX for comparison, are listed in Table 2 and Table 3, respectively. 

Generally, the target compounds **19a**–**m** have potent *in vitro* antibacterial activity against the tested Gram-positive strains (MICs: <0.008–32 µg/mL). Among of them, compounds **19h**, **19j**, **19k** and **19m** show good potency in inhibiting the growth of Methicillin-susceptible *Staphylococcus aureus* (MSSA), MRSA, methicillin-susceptible *Staphylococcus epidermidis* (MSSE), methicillin-resistant *Staphylococcus epidermidis* (MRSE) and *Streptococcus pneumonia* (MICs: <0.008–4 µg/mL). Notably, the four target compounds **19h**, **19j**, **19k** and **19m** have also useful activity (MICs: <0.008– 4 µg/mL) against both CPFX- and LVFX-resistant MRSA12-1/12-2 (MICs: 32->128 µg/mL) and MRSE12-3 (MICs: 16–32 µg/mL), CPFX-resistant MRSA12-3 (MIC: 64 µg/mL), as well as LVFX-resistant MSSA12-3 (MIC: 16 µg/mL) and MRSA12-4 (MIC: 32 µg/mL).

On the other hand, the target compounds **19a**–**m** are generally less active than the parent IMB-070593, CPFX and LVFX against the tested Gram-negative strains with few exceptions. It is noted that compounds **19e** and **19h** possess good potency against all of the four clinical strains of *Klebsiella pneumonia* (MICs: <0008–0.5 µg/mL). However, all of **19a**–**m**, like the three reference drugs, have virtually no activity against the extended-spectrum β-lactamase (ESBLs)-producing *Escherichia coli* and *Klebsiella pneumonia*, due partly to resistance of these ESBLs-producing strains inherent to fluoroquinolones.

Variations (R) on the benzene ring of the benzyl moiety in this study include mono-/bi-methoxyl, 3′,4′-methylenedioxyl/ethylenedioxyl, fluorine, chlorine and bromine substitution (Table 1). The activity imparted to IMB-070593 derivatives by R groups against Gram-positive strains was in the order: 4′-fluoro > 4′-chloro (cf. **19k** *vs.* **19l**) or 4′-methoxyl ≥ 2′-methoxyl > 3′-methoxyl (**19j** *vs.* **19h** *vs.* **19i**) for mono-substitution. However, introduction of an additional bromine atom at the 4′ position of 2′-methoxybenzyl moiety goes against the activity (**19h** *vs.* **19a**). In addition, compound **19c** is found to have antibacterial activity comparable to **19d**–**f**, and the same result is obtained in comparisons of the activity of **19b** and **19g**, therefore, analogs containing a disubstituted benzyl moiety seem to possess similar antibacterial activity.

**Table 2:** *In vitro* antibacterial activity of compounds **19a**–**m** against Gram-positive strains.

Strains	Compd. MIC (µg/mL)															
	19a	19b	19c	19d	19e	19f	19g	19h	19i	19j	19k	19l	19m	IMB-070593	CPFX	LVFX
*S.a.* ATCC	0.06	<0.008	0.5	0.015	<0.008	0.125	1	0.125	<0.008	<0.008	<0.008	0.06	0.03	0.015	1	0.25
MSSA12-1	0.25	0.125	0.06	0.06	0.125	0.125	0.125	0.06	0.125	0.06	0.015	0.25	0.06	<0.008	0.5	0.125
MSSA12-2	0.06	0.015	0.06	0.125	0.25	0.125	0.125	0.03	0.125	0.125	0.03	0.06	0.06	0.015	0.5	0.03
MSSA12-3	4	2	2	0.25	4	2	2	2	0.125	2	0.5	4	1	1	0.25	16
MSSA12-4	0.125	0.03	0.03	0.06	<0.008	0.06	0.06	0.03	0.06	0.03	<0.008	0.06	0.03	<0.008	<0.008	0.015
MRSA12-1	8	4	4	8	8	8	4	4	4	4	4	8	4	2	>128	64
MRSA12-2	8	4	4	4	8	4	4	4	4	4	4	8	2	2	64	32
MRSA12-3	0.25	0.03	0.125	0.125	0.25	0.125	0.06	0.03	0.125	0.125	<0.008	0.015	0.03	0.015	64	0.03
MRSA12-4	8	4	4	4	8	4	4	4	4	4	4	8	4	2	0.5	32
MSSE12-1	0.125	0.25	0.125	0.25	0.25	0.25	0.25	0.25	0.125	0.125	0.03	0.25	0.125	0.015	0.5	0.25
MSSE12-2	0.5	0.25	0.25	0.25	0.25	0.25	0.25	0.25	0.25	0.125	0.03	0.5	0.125	0.03	2	0.25
MSSE12-3	0.5	0.25	0.25	0.25	0.25	0.25	0.25	0.125	0.25	0.125	0.03	0.5	0.25	0.06	2	0.03
MSSE12-4	0.125	0.06	0.015	0.03	<0.008	0.03	0.06	<0.008	0.03	0.125	<0.008	0.015	0.015	0.015	<0.008	0.03
MRSE12-1	8	16	16	32	8	16	16	4	16	4	4	32	4	1	0.015	0.03
MRSE12-2	2	2	2	8	2	0.5	1	2	4	1	4	8	2	1	1	<0.008
MRSE12-3	8	2	4	16	8	4	1	2	8	4	4	16	2	1	32	16
MRSE12-4	0.5	0.125	0.125	0.125	0.25	0.25	0.25	0.125	4	0.125	0.03	0.25	2	0.015	0.015	0.03
*S.p.* 12-7	0.5	0.125	0.06	0.125	0.25	0.25	0.25	0.125	0.03	0.25	0.03	0.125	0.125	0.03	0.015	0.03
*S.p.* 12-23	0.5	0.125	0.015	0.25	<0.008	0.5	0.25	0.25	0.125	0.06	0.015	0.25	0.06	0.015	<0.008	0.015

LVFX, levofloxacin; CPFX, ciprofloxacin; *S.a.* ATCC, *S.a.* ATCC25923; *S.a., Staphylococcus aureus*; MSSA, Methicillin-susceptible *Staphylococcus aureus*; MRSA, Methicillin-resistant *Staphylococcus aureus*; MSSE, Methicillin-susceptible *Staphylococcus epidermidis*; MRSE, Methicillin-resistant *Staphylococcus epidermidis*; *S.p., Streptococcus pneumonia*.

**Table 3:** *In vitro* antibacterial activity of compounds **19a**–**m** against Gram-negative strains.

Strains	Compd. MIC (µg/mL)															
	19a	19b	19c	19d	19e	19f	19g	19h	19i	19j	19k	19l	19m	IMB-070593	CPFX	LVFX
*E. co.* ATCC	8	1	0.5	4	<0.008	0.25	<0.008	<0.008	<0.008	2	1	0.25	2	0.06	<0.008	<0.008
*E. co.* 12-1	64	8	16	32	4	16	8	2	1	8	8	16	8	0.5	0.125	0.5
*E. co.* 12-2	>64	>128	>64	>128	>128	>128	>128	16	>128	64	>128	128	16	16	8	4
*E. co.* 12-3	>64	>128	>64	>128	>128	>128	>128	>64	>128	>64	>128	>128	>128	64	64	32
*E. co.* 12-4	>64	>128	>64	>128	>128	>128	>128	>64	>128	>64	>128	>128	>128	32	16	8
*E. co. ^a^*12-1	>64	>128	>64	>128	>128	>128	>128	>64	>128	>64	>128	>128	>128	16	32	16
*E. co. ^a^* 12-2	>64	>128	>64	>128	>128	>128	>128	>64	>128	>64	>128	>128	>128	32	64	16
*E. co. ^a^* 12-3	>64	>128	>64	>128	>128	>128	>128	>64	>128	>64	>128	>128	>128	64	64	32
*E. co. ^a^* 12-4	64	8	16	16	4	16	2	1	8	8	8	16	4	0.5	0.125	0.25
*K. p.* 12-1	16	1	1	16	<0.008	4	8	0.125	2	2	1	1	2	0.25	0.125	0.03
*K. p.* 12-2	16	4	2	16	0.5	16	8	0.125	4	4	4	1	4	0.5	0.015	0.06
*K. p.* 12-3	16	4	2	8	<0.008	8	8	0.5	2	2	4	1	4	0.25	0.06	0.06
*K. p.* 12-4	32	4	2	16	<0.008	8	8	0.5	2	4	4	1	4	0.5	0.015	0.06
*K. p. ^a^*12-1	>64	64	>64	128	128	128	64	64	64	64	64	64	32	4	0.5	1
*K. p. ^a^* 12-2	>64	64	>64	128	128	128	64	64	32	64	64	64	32	8	4	4
*K. p. ^a^* 12-3	>64	>128	>64	>128	>128	>128	>128	>64	>128	>64	>128	>128	>128	32	64	16
*K. p. ^a^* 12-4	>64	64	>64	128	>128	128	64	64	128	64	128	128	32	16	4	8
*K. p. ^a^* 12-5	>64	>128	>64	>128	>128	128	>128	>64	>128	>64	>128	>128	>128	32	32	16
*P. a.* ATCC	16	2	2	8	4	16	8	1	4	2	2	1	2	2	1	8
*P. a.* 12-12	64	16	32	64	8	64	16	32	32	8	16	32	16	2	0.125	0.25
*P. a.* 12-14	8	4	16	8	8	8	4	8	4	2	4	8	2	2	32	32

LVFX, levofloxacin; CPFX, ciprofloxacin; E. co. ATCC, E. co. ATCC25922; E. co., Escherichia coli; K.p., Klebsiella pneumoniae; P. a. ATCC, P. a. ATCC27853; P.a., Pseudomonas aeruginosa; ^a^ Extended-spectrum β-lactamase-producing.

## 3. Experimental

### 3.1. Chemistry

Melting points were determined in open capillaries and are uncorrected. ^1^H-NMR (400, 500 or 600 MHz) and ^13^C-NMR (100 or 150 MHz) spectra were recorded at 25 °C on Varian Mercury spectrometers. Chemical shifts (δ) are given in ppm relative to tetramethylsilane or the respective NMR solvent. Electrospray ionization (ESI) mass spectra and high resolution mass spectra (HRMS) were obtained on a MDSSCIEX Q-Tap mass spectrometer. The reagents were all of analytical grade or chemically pure. TLC was performed on silica gel plates (Merck, ART5554 60F254).

### 3.2. Synthesis

#### 3.2.1. General Procedure for the Synthesis of Substituted *O*-Benzylhydroxylamines **4a**–**m**

To a solution of benzaldehydes **1a-d** (100 mmol) dissolved in methanol (500 mL) was added sodium borohydride (200 mmol) at room temperature, and the mixture was stirred at the same temperature for 2 h and concentrated under reduced pressure. The residue was diluted with dichloromethane (500 mL) and washed with water, dried over anhydrous sodium sulfate, and concentrated under reduced pressure to give the corresponding phenylmethanols **2a**–**d** as colorless oils.

To a solution of the above obtained phanylmethanols **2a**–**d** or commercially available **2e**–**m** (60 mmol), 2-hydroxyisoindoline-1,3-dione (60 mmol) and triphenylphosphine (75 mmol) in tetrahydrofuran (300 mL) was added dropwise a solution of diethyl azodicarboxylate (75 mmol) in tetrahydrofuran (15 mL) at 0 °C over 0.5 h. The mixture was stirred at the same temperature for 1 h and concentrated under reduced pressure. The residue was dissolved in methanol (300 mL) and stirred at room temperature for 1 h and filtered to give **3a**–**m** as white solids. Next, to a stirred solution of **3a**–**m** (60 mmol) in dichloromethane (200 mL) was added dropwise hydrazine hydrate (120 mmol). The reaction mixture was stirred at room temperature for 3 h, and then filtered. The filtrate was washed successively with 2 mol/L sodium hydroxide solution (200 mL) and brine (200 mL), dried over anhydrous sodium sulfate and concentrated under reduced pressure to give the corresponding benzyloxyamines **4a**–**m** as colorless oils (56%–70%, from **2a**–**d**; 66%–78%, from **2e**–**m**).

*O-(4-Bromo-2-methoxybenzyl) hydroxylamine* (**4a**). The title compound was obtained from **3a** as a colorless oil (87%), ^1^H-NMR (400 MHz, DMSO-*d_6_*) δ (ppm): 7.53 (1H, d, *J* = 8.4 Hz, Ar-H), 7.21 (1H, d, *J* = 2.4 Hz, Ar-H), 6.98 (1H, q, *J_1_* = 8.4 Hz, *J_2_* = 2.4 Hz, Ar-H), 5.21 (2H, s, OCH_2_Ar), 3.79 (3H, s, O-CH_3_). MS-ESI (*m/z*): 233 (M+H)^+^.

*O-((2,3-Dihydrobenzo[b]*[1,4]*dioxin-6-yl)methyl) hydroxylamine* (**4b**). The title compound was obtained from **3b** as a colorless oil (85%), ^1^H-NMR (400 MHz, DMSO-*d_6_*) δ (ppm): 6.81–6.79 (2H, m, Ar-H), 6.77–6.75 (1H, m, Ar-H), 5.95 (2H, s, OCH_2_Ar), 4.21 (4H, s, 2 × OCH_2_). MS-ESI (*m/z*): 182 (M+H)^+^.

*O-(2,5-Dimethoxybenzyl) hydroxylamine* (**4c**). The title compound was obtained from **3c** as a colorless oil (90%), ^1^H-NMR (400 MHz, DMSO-*d_6_*) δ (ppm): 6.89 (2H, m, Ar-H), 6.81 (1H, m, Ar-H), 4.57 (2H, s, OCH_2_Ar), 3.71 (6H, s, O-CH_3_). MS-ESI (*m/z*): 184 (M+H)^+^.

*O-(3,5-Dimethoxybenzyl) hydroxylamine* (**4d**). The title compound was obtained from **3d** as a colorless oil (88%), ^1^H-NMR (400 MHz, DMSO-*d_6_*) δ (ppm): 6.46 (2H, s), 6.39 (1H, s), 4.50 (2H, s), 3.73 (6H, s). MS-ESI (*m/z*): 184 (M+H)^+^.

*O-(2,3-Dimethoxybenzyl) hydroxylamine* (**4e**). The title compound was obtained from **3e** as a colorless oil (91%), ^1^H-NMR (400 MHz, DMSO-*d_6_*) δ (ppm): 7.03 (1H, t, *J* = 7.8 Hz, Ar-H), 6.68 (1H, d, *J* = 7.8 Hz, Ar-H), 6.90 (1H, d, *J* = 7.8 Hz, Ar-H), 4.56 (2H, s, OCH_2_Ar), 3.78 (3H, s, O-CH_3_), 3.69 (3H, s, O-CH_3_). MS-ESI (*m/z*): 184 (M+H)^+^.

*O-(3,4-Dimethoxybenzyl) hydroxylamine* (**4f**). The title compound was obtained from **3f** as a colorless oil (89%), ^1^H-NMR (400 MHz, DMSO-*d_6_*) δ (ppm): 7.20 (1H, m, Ar-H), 6.93 (2H, m, Ar-H), 5.05 (2H, s, OCH_2_Ar), 3.78 (6H, s, O-CH_3_). MS-ESI (*m/z*): 184 (M+H)^+^.

*O-(Benzo[d]*[1,3]*dioxol-5-ylmethyl) hydroxylamine* (**4g**). The title compound was obtained from **3g** as a colorless oil (84%), ^1^H-NMR (400 MHz, DMSO-*d_6_*) δ (ppm): 6.98 (1H, d, *J* = 1.2 Hz, Ar-H), 6.94 (1H, d, *J* = 8 Hz, Ar-H), 6.91 (1H, q, *J_1_* = 8 Hz, *J_2_* = 1.2 Hz, Ar-H), 6.05 (2H, s, OCH_2_Ar), 4.91 (2H, s, OCH_2_O). MS-ESI (*m/z*): 168 (M+H)^+^.

*O-(2-Methoxybenzyl) hydroxylamine* (**4h**). The title compound was obtained from **3h** as a colorless oil (85%), ^1^H-NMR (600 MHz, DMSO-*d_6_*) δ (ppm): 7.26 (2H, m, Ar-H), 6.95 (1H, d, *J* = 7.8 Hz, Ar-H), 6.91 (1H, *J* = 7.8 Hz, Ar-H), 4.56 (2H, s, Ar-H), 3.76 (3H, s, O-CH_3_). MS-ESI (*m/z*): 154 (M+H)^+^.

*O-(3-Methoxybenzyl) hydroxylamine* (**4i**). The title compound was obtained from **3i** as a colorless oil (86%), ^1^H-NMR (400 MHz, DMSO-*d_6_*) δ (ppm): 7.33 (1H, m, Ar-H), 6.97 (3H, m, Ar-H) 5.00 (2H, s, OCH_2_Ar), 3.77 (3H, s, O-CH_3_). MS-ESI (*m/z*): 154 (M+H)^+^.

*O-(4-Methoxybenzyl) hydroxylamine* (**4j**). The title compound was obtained from **3j** as a colorless oil (88%), ^1^H-NMR (400 MHz, DMSO-*d_6_*) δ (ppm): 7.34 (2H, d, *J* = 8.4 Hz, Ar-H), 6.97 (2H, d, *J* = 8.4 Hz, Ar-H), 4.90 (2H, s, OCH_2_Ar), 3.77 (3H, s, O-CH_3_). MS-ESI (*m/z*): 154 (M+H)^+^.

*O-(4-Fluorobenzyl) hydroxylamine* (**4k**). The title compound was obtained from **3k** as a colorless oil (84%), ^1^H-NMR (400 MHz, DMSO-*d_6_*) δ (ppm): 7.35 (2H, m, Ar-H), 7.16 (2H, m, Ar-H), 4.54 (2H, s, OCH_2_Ar). MS-ESI (*m/z*): 142 (M+H)^+^.

*O-(4-Chlorobenzyl) hydroxylamine* (**4l**). The title compound was obtained from **3l** as a colorless oil (90%), ^1^H-NMR (400 MHz, DMSO-*d_6_*) δ (ppm): 7.38 (2H, d, *J* = 8.4 Hz, Ar-H), 7.34 (2H, d, *J* = 8.4 Hz, Ar-H), 4.56 (2H, s, OCH_2_Ar). MS-ESI (*m/z*): 158 (M+H)^+^.

*O-Benzyl hydroxylamine* (**4m**). The title compound was obtained from **3m** as a colorless oil (92%), ^1^H-NMR (400 MHz, DMSO-*d_6_*) δ (ppm): 7.39 (4H, m, Ar-H), 7.33 (1H, m, Ar-H), 5.12 (2H, s, OCH_2_Ar). MS-ESI (*m/z*): 124 (M+H)^+^.

*tert-Butyl 3-cyano-4-(methoxyimino)piperidine-1-carboxylate* (**6**). To a solution of hydroxylamine hydrochloride (5.01 g, 60 mmol) in methanol (150 mL) was added sodium hydroxide (2.40 g, 60 mmol), and the mixture stirred at room temperature for 30 minutes. The piperidinone **5** (11.18 g, 50 mmol) was added to the mixture, stirred at 50 °C for 3 h and then filtered. The filtrate was concentrated under reduced pressure. The residue was diluted with ethyl acetate (200 mL), washed with water, dried over anhydrous sodium sulfate, and concentrated under reduced pressure to afford the title compound **6** (14.12 g, 93%) as a yellow oil. ^1^H-NMR (400 MHz, DMSO-*d_6_*) δ (ppm): 4.48 (1H, s, piperidine-H), 4.25 (1H, m, piperidine-H), 4.04 (1H, m, piperidine-H), 3.84 (3H, s, NOCH_3_), 3.14 (1H, brs, piperidine-H), 2.93 (1H, brs, piperidine-H), 2.41 (2H, m, piperidine-H), 1.42 (9H, s, Boc-9H). MS-ESI (*m/z*): 254 (M+H)^+^.

*tert-Butyl 3-carbamoyl-4-(methoxyimino)piperidine-1-carboxylate* (**7**). To a solution of **6** (10.12 g, 40 mmol) and potassium carbonate (11.06 g, 80 mmol) in dimethyl sulfoxide (100 mL) was added hydrogen peroxide (36.27 mL, 320 mmol) at 15 °C for 1 h, and stirred at room temperature for 3 h. The mixture was diluted with water (200 mL), and extracted by ethyl acetate (200 mL). The combined extracts were washed with brine, dried over anhydrous sodium sulfate, and then concentrated under reduced pressure to give the title compound **7** (7.71 g, 71%) as a light yellow oil. ^1^H-NMR (400 MHz, DMSO-*d_6_*) δ (ppm): 7.37 (1H, s, CONH_2_), 7.00 (1H, s, CONH_2_), 4.19–4.10 (1H, brs, piperidine-H), 4.00–3.92 (1H, m, piperidine-H), 3.84 (1H, s, piperidine-H), 3.72 (3H, s, NOCH_3_), 3.11 (1H, m, piperidine-H), 2.61–2.53 (1H, m, piperidine-H), 2.24 (1H, brs, piperidine-H), 1.42 (1H, s, piperidine-H), 1.38 (9H, s, Boc-9H). MS-ESI (*m/z*): 294 (M+Na)^+^.

*tert-Butyl 3-amino-4-(methoxyimino)piperidine-1-carboxylate* (**8**). To a stirred solution of **7** (13.55 g, 50 mmol) in acetonitrile (350 mL) was added dropwise 10% sodium hypobromite solution (106.20 mL, 90 mmol) at 5 °C for 1 h. The reaction mixture was stirred at room temperature for 10 h, and adjusted to pH 6.5–7 with 20% acetic acid, and then concentrated under reduced pressure. The residue was dissolved in water (200 mL), adjusted to pH 4–3 with 10% hydrochloride and washed with ethyl acetate. The water layer was adjusted to pH 9 with 15% sodium hydroxide and extracted with ethyl acetate. The combined extracts were dried over anhydrous sodium sulfate and concentrated under reduced pressure to yield the title compound **8** (7.65 g, 63%) as a yellow oil. ^1^H-NMR (400 MHz, DMSO-*d_6_*) δ (ppm): 4.09–4.02 (1H, m, piperidine-H), 3.74 (3H, s, NOCH_3_), 3.57 (1H, m, piperidine-H), 3.31–3.25 (1H, m, piperidine-H), 3.11 (1H, s, piperidine-H), 2.99–2.92 (1H, brs, piperidine-H), 2.71–2.66 (1H, m, piperidine-H), 1.98 (1H, brs, piperidine-H), 1.38 (9H, s, Boc-9H). MS-ESI (*m/z*): 244 (M+H)^+^.

*3-Amino-4-methoxyiminopiperidine dihydrocholoride* (**9**). To a solution of **8** (7.29 g, 30 mmol) in dichloromethane was pumped dried hydrochloride gas at room temperature for 30 minutes. The mixture was stirred for another 1 h at room temperature, and concentrated under reduced pressure. The residue was treated with ethyl acetate. The precipitate was collected by suction, and dried under vacuum to afford the title compound **9** (2.15 g, 52%) as a white solid, m.p.: 224–226 °C.^ 1^H-NMR (300 MHz, D_2_O) δ (ppm): 4.45–4.50 (1H, m, piperidine-H), 4.01–3.97 (4H, m, piperidine-H, NOCH_3_), 3.70–3.65 (1H, m, piperidine-H), 3.59–3.53 (1H, m, piperidine-H), 3.34–3.28 (1H, m, piperidine-H), 3.23–3.15 (1H, m, piperidine-H), 2.46–2.37 (1H, m, piperidine-H). M.p.: 178–181 °C. MS-FAB (*m/z*): 144 (M+H)^+^.

*(9H-Fluoren-9-yl)methyl 3-[(9H-fluoren-9-yl)methoxycarbonylamino]-4-(methoxyimino)piperidine-1-carboxylate* (**10**). To a stirring solution of **9** (3.24g, 15 mmol) and triethylamine (6.24 mL,45 mmol) in dichloromethane (300 mL) was added in portions 9-fluorenylmethyl chloroformate (7.76 g, 30 mmol) at 0 °C, and the mixture stirred at room temperature for 5 h, then washed with water, dried over anhydrous sodium sulfate, and concentrated under reduced pressure. The crude product was purified by column chromatography (silica gel), eluting with petroleum ether and ethyl acetate (v:v = 3:1) to produce the title compound **10** (5.02 g, 57%) as an off-white solid, m.p.: 152–154 °C.^ 1^H-NMR (400 MHz, CDCl_3_) δ (ppm): 7.76 (4H, m, Fmoc-ArH), 7.61 (4H, m, Fmoc-ArH), 7.39 (4H, m, Fmoc-ArH), 7.32 (4H, m, Fmoc-ArH), 4.44 (4H, d, *J* = 6.8 Hz, 2 × Fmoc-CH_2_), 4.27 (2H, m, 2 × Fmoc-CH), 4.12 (1H, m, piperidine-H), 3.92 (3H, s, NOCH_3_), 3.20 (1H, brs, piperidine-H), 3.04 (1H, t, *J* = 8.8 Hz, piperidine-H), 2.89 (1H, t, *J* = 11 Hz, piperidine-H), 2.10 (2H, brs, piperidine-H), 1.25 (1H, m, piperidine-H). MS-ESI (*m/z*): 610 (M+Na)^+^.

*(9H-Fluoren-9-yl)methyl 3-[(9H-fluoren-9-yl)methoxycarbonylamino]-4-oxopiperidine-1-carboxylate* (**11**). To a solution of **10** (11.75 g, 20 mmol) and ethyl acetoacetate (26.02 g, 200 mmol) in methanol (200 mL) was added concentrated hydrochloride (0.4 mmol, 33.33 mL) and stirred at 60 °C for 6 h. The mixture was concentrated under reduced pressure. The residue was dissolved in water (200 mL), adjusted to pH 6.5–7 with saturated sodium bicarbonate solution, and extracted with dichloromethane. The combined extracts were dried over anhydrous sodium sulfate and concentrated under reduced pressure. The crude product was purified by column chromatography (silica gel), eluting with petroleum ether and ethyl acetate (v:v = 2:1) to afford the title compound **11** (5.81 g, 52%) as a white solid, m.p.: 141–142 °C.^ 1^H-NMR (600 MHz, CDCl_3_) δ (ppm): 7.77 (4H, m, Fmoc-ArH), 7.61 (4H, m, Fmoc-ArH), 7.41 (4H, m, Fmoc-ArH), 7.31 (4H, m, Fmoc-ArH), 4.63 (4H, s, 2 × Fmoc-CH_2_), 4.28 (2H, m, 2 × Fmoc-CH), 4.12 (1H, m, piperidine-H), 3.72 (1H, brs, piperidine-H), 3.08 (1H, brs, piperidine-H), 2.84 (1H, brs, piperidine-H), 2.66–2.59 (2H, m, piperidine-H), 1.26 (1H, m, piperidine-H). MS-ESI (*m/z*): 581 (M+Na)^+^.

*(9H-Fluoren-9-yl)methyl 3-[(9H-fluoren-9-yl)methoxycarbonylamino]-4-benzyloxyiminopiperidine-1-carboxylate* (**12**). To a solution of **11** (5.58 g, 10 mmol) in ethanol (100 mL) was added **4m** (2.46 g, 20 mmol) and the mixture stirred at room temperature for 10 h and concentrated under reduced pressure. The residue was dissolved in ethyl acetate (100 mL) and washed with brine (200 mL). The combined extracts were dried over anhydrous sodium sulfate and concentrated under reduced pressure. The crude product was purified by column chromatography (silica gel), eluting with petroleum ether acetate (v:v = 2:1) to yield the title compound **12** (4.95 g, 75%) as a white solid, m.p.: 169–171 °C. ^1^H-NMR (600 MHz, CDCl_3_) δ (ppm): 7.75 (4H, m, Fmoc-ArH), 7.61 (4H, m, Fmoc-ArH), 7.42–7.37 (9H, m, Fmoc-ArH, Ar-H), 7.31 (4H, m, Fmoc-ArH), 5.15 (2H, s, OCH_2_Ar), 4.43 (4H, s, 2 × Fmoc-CH_2_), 4.26 (2H, m, 2 × Fmoc-CH), 4.18 (1H, m, piperidine-H), 3.28 (1H, brs, piperidine-H), 3.00 (1H, m, piperidine-H), 2.85 (1H, m, piperidine-H), 2.15 (1H, brs, piperidine-H), 1.53 (2H, m, piperidine-H). MS-ESI (*m/z*): 664 (M+H)^+^, 686 (M+Na)^+^, 702 (M+K)^+^.

*3-Amino-4-benzyloxyiminopiperidine dihydrocholoride* (**13m**). To a solution of **12** (2 g, 3 mmol) in tetrahydrofuran (50 mL) was added 10% sodium hydroxide (6 mL, 15 mmol) and stirred at 60 °C for 20 h. The mixture was concentrated under reduced pressure. The residue was treated with dichloromethane (100 mL) and washed with water. The organic layer was dried over anhydrous sodium sulfate and filtered. To the filtrate was pumped dried hydrochloride gas at room temperature for 30 minutes and concentrated under reduced pressure to afford the title compound **13m** (0.3 g, 45%) as a white solid, m.p.: 153–155 °C. ^1^H-NMR (600 MHz, DMSO-*d_6_*) δ (ppm): 7.39 (4H, m), 7.33 (1H, m, Ar-H), 5.12 (2H, s, OCH_2_Ar), 3.95 (1H, m, piperidine-H), 3.64 (1H, m, piperidine-H), 3.07 (1H, m, piperidine-H), 2.97 (1H, m, piperidine-H), 2.88 (1H, m, piperidine-H), 2.78 (1H, m, piperidine-H), 2.38 (1H, m, piperidine-H). MS-ESI (*m/z*): 220 (M+H)^+^, 242 (M+Na)^+^. M.p.: 153–155 °C.

#### 3.2.2. General Procedure for the Synthesis of 3-Amino-4-(substituted benzyloxyimino)piperidine Dihydrocholorides **13a**–**l**

To a solution of **5** (70 mmol) in ethanol (100 mL) was added **4a**–**l** (140 mmol) and stirred at room temperature for 10 h and concentrated under reduced pressure. The residue was dissolved in ethyl acetate (200 mL) and washed with brine (200 mL). The organic layer was dried over anhydrous sodium sulfate and concentrated under reduced pressure to afford compounds **14a**–**l** as yellow oils.

To a solution of **14a**–**l** (50 mmol) and potassium carbonate (100 mmol) in dimethyl sulfoxide (150 mL) was added hydrogen peroxide (400 mmol) at 15 °C for 1 h, and stirred at room temperature for 3 h. The mixture was diluted with water (300 mL) and extracted by ethyl acetate (400 mL). The combined extracts were washed with brine (400 mL), dried over anhydrous sodium sulfate, and then concentrated under reduced pressure to give compounds **15a**–**l** as colorless or light yellow oils.

To a stirred solution of **15a**–**l** (50 mmol) in acetonitrile (350 mL) was added dropwise 10% sodium hypobromite solution (90 mmol) at 5 °C for 1 h. The reaction mixture was stirred at room temperature for 10 h, adjusted to pH 6.5–7 by 20% acetic acid and then concentrated under reduced pressure. The residue was dissolved in water (200 mL), adjusted to pH 4 with 10% hydrochloride and washed by ethyl acetate. The water layer was adjusted to pH 9 with 10% sodium hydroxide and extracted by ethyl acetate. The combined extracts were dried over anhydrous sodium sulfate, and concentrated under reduced pressure to afford compounds **16a**–**l** as yellow oils.

To a solution of **16a**–**l** (7.29 g, 30 mmol) in dichloromethane was pumped dried hydrochloride gas at room temperature for 30 minutes. The mixture was stirred for another 1 h at room temperature, and concentrated under reduced pressure. The residue was treated with ethyl acetate. The precipitate was collected by suction, and dried under vacuum to afford compounds **13a**–**l** (19%–26%, from **5**) as white or yellow solids.

*3-Amino-4-(4′-bromo-2′-methoxybenzyloxyimino)piperidine dihydrocholoride* (**13a**). The title compound was obtained from **16a** as an off-white solid easily absorbing moisture (50%).

*3-Amino-4-(3′,4′-ethylenedioxylbenzyloxyimino)piperidine dihydrocholoride* (**13b**). The title compound was obtained from **16b** as a white solid (51%), m.p.: 190–192 °C. ^1^H-NMR (400 MHz, DMSO-*d_6_*) δ (ppm): 6.87 (3H, m, Ar-H), 5.02 (2H, s, OCH_2_Ar), 4.41 (1H, m, piperidine-H), 4.23 (4H, s, 2 × OCH_2_), 3.71 (1H, m, piperidine-H), 3.53 (1H, m, piperidine-H), 3.37 (1H, m, piperidine-H), 3.23–3.11 (2H, m, piperidine-H), 2.98 (1H, m, piperidine-H). MS-ESI (*m/z*): 278 (M+H)^+^. 

*3-Amino-4-(2′,5′-dimethoxylbenzyloxyimino)piperidine dihydrocholoride* (**13c**). The title compound was obtained from **16c** as a light yellow solid (53%), m.p.: 189–192 °C. ^1^H-NMR (400 MHz, D_2_O) δ (ppm): 7.07 (1H, m, Ar-H), 7.02 (2H, m, Ar-H), 5.20 (2H, s, OCH_2_Ar), 4.50 (1H, *J_1_* = 12.4 Hz, *J_2_* = 5.6 Hz, piperidine-H), 4.00 (1H, m, piperidine-H), 3.84 (3H, s, OCH_3_), 3.82 (3H, s, OCH_3_), 3.67 (1H, m, piperidine-H), 3.56 (1H, m, piperidine-H), 3.31 (1H, t, *J* = 12.4 Hz, piperidine-H), 3.18 (1H, m, piperidine-H), 2.44 (1H, m, piperidine-H). MS-ESI (*m/z*): 280 (M+H)^+^. 

*3-Amino-4-(3′,5′-dimethoxylbenzyloxyimino)piperidine dihydrocholoride* (**13d**). The title compound was obtained from **16d** as a white solid (50%), m.p.: 212–213 °C. ^1^H-NMR (400 MHz, DMSO-*d_6_*) δ (ppm): 6.55 (2H, m, Ar-H), 6.45 (1H, m, Ar-H), 5.09 (2H, s, OCH_2_Ar), 4.45 (1H, m, piperidine-H), 3.75 (6H, s, OCH_3_), 3.72 (1H, m, piperidine-H), 3.38 (1H, m, piperidine-H), 3.26 (1H, m, piperidine-H), 3.17 (1H, m, piperidine-H), 2.98 (1H, m, piperidine-H), 2.58 (1H, m, piperidine-H). MS-ESI (*m/z*): 280 (M+H)^+^, 302 (M+Na)^+^.

*3-Amino-4-(2′,3′-dimethoxylbenzyloxyimino)piperidine dihydrocholoride* (**13e**). The title compound was obtained from **16e** as a light yellow solid (54%), m.p.: 194–196 °C. ^1^H-NMR (600 MHz, DMSO-*d_6_*) δ (ppm): 7.07 (2H, m, Ar-H), 6.98 (1H, m, Ar-H), 5.15 (2H, s, OCH_2_Ar), 4.44 (1H, m, piperidine-H), 3.81 (3H, s, OCH_3_), 3.74 (3H, s, OCH_3_), 3.70 (1H, m, piperidine-H), 3.36 (1H, m, piperidine-H), 3.23 (1H, m, piperidine-H), 3.16 (1H, m, piperidine-H), 2.98 (1H, m, piperidine-H), 2.58 (1H, m, piperidine-H). MS-ESI (*m/z*): 280 (M+H)^+^.

*3-Amino-4-(3′,4′-dimethoxylbenzyloxyimino)piperidine dihydrocholoride* (**13f**). The title compound was obtained from **16f** as a light yellow solid (52%), m.p.: 183–184 °C. ^1^H-NMR (600 MHz, DMSO-*d_6_*) δ (ppm): 6.95 (3H, m, Ar-H), 5.05 (2H, s, OCH_2_Ar), 4.45 (1H, m, piperidine-H), 3.75 (6H, s, OCH_3_), 3.37 (1H, m, piperidine-H), 3.28 (1H, m, piperidine-H), 3.13 (1H, m, piperidine-H), 2.96 (1H, m, piperidine-H), 2.55 (1H, m, piperidine-H), 1.21 (1H, m, piperidine-H). MS-ESI (*m/z*): 280 (M+H)^+^. 

*3-Amino-4-(3′,4′-methylenedioxybenzyloxyimino)piperidine dihydrocholoride* (**13g**). The title compound was obtained from **16e** as a white solid (50%), m.p.: 199–201 °C. ^1^H-NMR (600 MHz, DMSO-*d_6_*) δ (ppm): 7.01 (1H, s, Ar-H), 6.89 (2H, m, Ar-H), 6.02 (2H, s, OCH_2_Ar), 5.04 (2H, s, 2 × OCH_2_), 4.36 (1H, m, piperidine-H), 3.68 (1H, brs, piperidine-H), 3.35 (1H, m, piperidine-H), 3.22 (1H, m, piperidine-H), 3.12 (1H, m, piperidine-H), 2.98 (1H, m, piperidine-H), 2.48 (1H, m, piperidine-H). MS-ESI (*m/z*): 264 (M+H)^+^, 527 (2M+H)^+^. 

*3-Amino-4-(2′-methoxylbenzyloxyimino)piperidine dihydrocholoride* (**13h**). The title compound was obtained from **16e** as a light yellow solid (55%), m.p.: 199–200 °C. ^1^H-NMR (400 MHz, D_2_O) δ (ppm): 7.44 (2H, m, Ar-H), 7.13 (1H, d, *J* = 8.4 Hz, Ar-H), 7.07 (1H, t, *J* = 7.6 Hz, Ar-H), 5.24 (2H, s, OCH_2_Ar), 4.44 (1H, q, *J_1_* = 12 Hz, *J_2_* = 5.2 Hz, piperidine-H), 3.96 (1H, q, *J_1_* = 12 Hz, *J_2_* = 5.2 Hz, piperidine-H), 3.88 (3H, s, OCH_3_), 3.63 (1H, q, *J_1_* = 12.8 Hz, *J2* = 5.6 Hz, piperidine-H), 3.53 (1H, m, piperidine-H), 3.25 (1H, t, *J* = 12 Hz, piperidine-H), 3.13 (1H, m, piperidine-H), 2.41 (1H, m, piperidine-H). MS-ESI (*m/z*): 250 (M+H)^+^. 

*3-Amino-4-(3′-methoxylbenzyloxyimino)piperidine dihydrocholoride* (**13i**). The title compound was obtained from **16e** as a light yellow solid (51%), m.p.: 215–217 °C. ^1^H-NMR (400 MHz, D_2_O) δ (ppm): 7.43 (1H, m, Ar-H), 7.04 (3H, m, Ar-H), 5.15 (2H, s, OCH_2_Ar), 4.38 (1H, m, piperidine-H), 3.91 (1H, m, piperidine-H), 3.85 (3H, s, OCH_3_), 3.62–3.52 (2H, m, piperidine-H), 3.24–3.17 (1H, m, piperidine-H), 3.14–3.07 (1H, m, piperidine-H), 2.42–2.34 (1H, m, piperidine-H).MS-ESI (*m/z*): 250 (M+H)^+^. 

*3-Amino-4-(4′-methoxylbenzyloxyimino)piperidine dihydrocholoride* (**13j**). The title compound was obtained from **16e** as an off-white solid (53%), m.p.: 221–222 °C. ^1^H-NMR (600 MHz, DMSO-*d_6_*) δ (ppm): 7.35 (2H, d, *J* = 9 Hz, Ar-H), 6.93 (2H, *J* = 9 Hz, Ar-H), 5.07 (2H, s, OCH_2_Ar), 4.41 (1H, m, piperidine-H), 3.76 (3H, s, OCH_3_), 3.71 (1H, m, piperidine-H), 3.38 (1H, m, piperidine-H), 3.26 (1H, m, piperidine-H), 3.20 (1H, m, piperidine-H), 3.12 (1H, m, piperidine-H), 2.95 (1H, m, piperidine-H). MS-ESI (*m/z*): 250 (M+H)^+^. 

*3-Amino-4-(4′-flourobenzyloxyimino)piperidine dihydrocholoride* (**13k**). The title compound was obtained from **16e** as a yellow solid as yellow solid easily absorbing moisture (50%).

*3-Amino-4-(4′-chlorobenzyloxyimino)piperidine dihydrocholoride* (**13l**). The title compound was obtained from **16e** as a white solid (56%), m.p.: 200–202 °C. ^1^H-NMR (400 MHz, DMSO-*d_6_*) δ (ppm): 7.44 (4H, m, Ar-H), 5.14 (2H, s, OCH_2_Ar), 4.43 (1H, m, piperidine-H), 3.72 (1H, m, piperidine-H), 3.38 (1H, m, piperidine-H), 3.24 (1H, m, piperidine-H), 3.16 (1H, m, piperidine-H), 2.98 (1H, m, piperidine-H), 2.57 (1H, m, piperidine-H). MS-ESI (*m/z*): 254 (M+H)^+^.

#### 3.2.3. General Procedure for the Synthesis of 7-[3-Amino-4-(substituted benzyloxyimino)piperidin-1-yl]-1-cyclopropyl-6-fluoro-8-methoxy-4-oxo-1,4-dihydroquinoline-3-carboxylic Acids **19a**–**m**

A mixture of boric acid (9.27 g, 150 mmol) and acetic anhydride (54.06 g, 530 mmol) was stirred at 110 °C for 1.5 h, added acetic acid (50 mL) and then stirred for 1h at the same temperature. To the reaction mixture temperature was added **17** (32.3 g, 100 mmol) at 95 °C and stirred at the same temperature for 2 h. After cooling to room temperature, the mixture was poured into ice water (500 mL) slowly and stirred for 0.5 h. The resulting solid was collected by suction and washed successively with water (50 mL), chilled ethanol (50 mL) and ethyl ether (50 mL), and dried under vacuum to afford compound **18** (29.4 g, 69%) as a white solid, m.p.: 195–196 °C.

To a solution of **13a**–**m** (1 mmol) and triethylamine (3 mmol) in acetonitrile (10 mL) was added **18** (0.8 mmol) at room temperature. The reaction mixture was stirred overnight at 50 °C, and concentrated under reduced pressure. The residue was dissolved in a solution of 5% sodium hydroxide solution (8 mL) and stirred for 1 h at 50 °C. After cooling to room temperature, the mixture was adjusted to pH 7.0–7.5 with 5% acetic acid, and extracted with dichloromethane. The combined extracts were concentrated under reduced pressure. The residue was dissolved in 20% acetic acid (10 mL), stirred for 0.5 h at 50 °C, and filtered. The filtrate was adjusted to pH 6.5–7.5 with 15% sodium hydroxide and extracted by dichloromethane. The combined extracts were dried over anhydrous sodium sulfate and concentrated under reduced pressure. The crude product was purified by column chromatography (silica gel), eluting with dichloromethane and methanol (v:v = 10:1) to afford the target compounds **19a**–**m** as off-white or yellow solids. 

*7-[3-Amino-4-(4′-bromo-2′-methoxybenzyloxyimino)piperidin-1-yl]-1-cyclopropyl-6-fluoro-8-methoxy-4-oxo-1,4-dihydroquinoline-3-carboxylic acid* (**19a**). Prepared from **13a** as an off-white solid (19%), m.p.: >230 °C. ^1^H-NMR (400 MHz, DMSO-*d_6_*) δ (ppm): 8.74 (1H, s, C_2_-H), 7.65 (1H, d, *J* = 12 Hz, C_5_-H), 7.42 (1H, d, *J* = 8 Hz, Ar-H), 7.22 (1H, d, *J* = 2 Hz, Ar-H), 6.97 (1H, q, *J_1_* = 8 Hz, *J_2_* = 6 Hz, Ar-H), 5.10 (2H, s, O-CH_2_Ar), 4.86 (1H, m, cyclopropyl CH), 4.45 (1H, m, piperidine-H), 4.15 (1H, m, piperidine-H), 3.68 (6H, s, O-CH_3_), 2.96 (1H, m, piperidine-H), 2.85 (1H, m, piperidine-H), 2.77 (1H, m, piperidine-H), 2.68 (1H, m, piperidine-H), 2.40 (1H, m, piperidine-H), 0.93 (4H, m, 2 × cyclopropyl CH_2_). MS-ESI (*m/z*): 603 (M+H)^+^. HRMS-ESI (*m/z*): Calcd. for C_27_H_29_O_6_N_4_FBr (M+H)^+^: 603.1254; Found 603.1276.

*7-[3-Amino-4-(3′,4′-ethylenedioxybenzyloxyimino)piperidin-1-yl]*-*1-cyclopropyl-6-fluoro-8-methoxy-4-oxo-1,4-dihydroquinoline-3-carboxylic acid* (**19b**). Prepared from **13b** as an off-white solid (33%), m.p.: 78–81 °C. ^1^H-NMR (400 MHz, DMSO-*d_6_*) δ (ppm): 8.70 (1H, s, C_2_-H), 7.77 (1H, d, *J* = 12 Hz, C_5_-H), 6.87 (1H, s, Ar-H), 6.83 (2H, m, Ar-H), 4.96 (2H, s, O-CH_2_Ar), 4.80 (1H, m, cyclopropyl CH), 4.42 (1H, m, piperidine-H), 4.23 (4H, s, 2 × OCH_2_), 3.74 (3H, s, O-CH_3_), 3.40 (1H, m, piperidine-H), 3.19 (1H, m, piperidine-H), 3.03 (1H, m, piperidine-H), 2.89 (1H, m, piperidine-H), 2.72 (1H, m, piperidine-H), 2.33 (1H, m, piperidine-H), 1.05 (4H, m, 2 × cyclopropyl CH_2_). MS-ESI (*m/z*): 553 (M+H)^+^, 555 (M+Na)^+^. HRMS-ESI (*m/z*): Calcd. for C_28_H_30_O_7_N_4_F (M+H)^+^: 553.2093; Found 553.2109. M.p.: 78–81 °C.

*7-[3-Amino-4-(2′,5′-dimethoxybenzyloxyimino)piperidin-1-yl]-1-cyclopropyl-6-fluoro-8-methoxy-4-oxo-1,4-dihydroquinoline-3-carboxylic acid* (**19c**). Prepared from **13c** as an off-white solid (21%), m.p.: 80–81 °C. ^1^H-NMR (400 MHz, DMSO-*d_6_*) δ (ppm): 8.62 (1H, s, C_2_-H), 7.78 (1H, d, *J* = 12 Hz, C_5_-H), 6.94 (1H, d, *J* = 8 Hz, Ar-H), 6.87 (1H, q, *J_1_* = 8H, *J_2_* = 3 Hz, Ar-H), 6.84 (1H, d, *J* = 3 Hz, Ar-H), 5.08 (2H, s, O-CH_2_Ar), 4.93 (1H, m, cyclopropyl CH), 4.42 (1H, m, piperidine-H), 4.17 (1H, m, piperidine-H), 3.69 (9H, s, O-CH_3_), 3.17 (1H, m, piperidine-H), 3.11 (1H, m, piperidine-H), 2.91–2.86 (1H, m, piperidine-H), 2.75 (1H, m, piperidine-H), 2.39 (1H, m, piperidine-H), 0.98 (4H, m, 2 × cyclopropyl CH_2_). MS-ESI (*m/z*): 555 (M+H)^+^. HRMS-ESI (*m/z*): Calcd. for C_28_H_32_O_7_N_4_F (M+H)^+^: 555.2249; Found 555.2252. M.p.: 80–81 °C.

*7-[3-Amino-4-(3′,5′-dimethoxybenzyloxyimino)piperidin-1-yl]-1-cyclopropyl-6-fluoro-8-methoxy-4-oxo-1,4-dihydroquinoline-3-carboxylic acid* (**19d**). Prepared from **13d** as an off-white solid (23%), m.p.: 68–70 °C. ^1^H-NMR (400 MHz, DMSO-*d_6_*) δ (ppm): 8.70 (1H, s, C_2_-H), 7.78 (1H, d, *J* = 12 Hz, C_5_-H), 6.52 (1H, s, Ar-H), 6.42 (1H, s, Ar-H), 6.25 (1H, s, Ar-H), 5.03 (2H, s, O-CH_2_Ar), 4.87 (1H, m, cyclopropyl CH), 4.41 (1H, m, piperidine-H), 4.16 (1H, m, piperidine-H), 3.67 (9H, s, O-CH_3_), 3.43 (1H, m, piperidine-H), 3.18 (1H, m, piperidine-H), 2.88 (1H, m, piperidine-H), 2.75 (1H, m, piperidine-H), 2.41 (1H, m, piperidine-H), 1.03 (4H, m, 2 × cyclopropyl CH_2_). MS-ESI (*m/z*): 555 (M+H)^+^. HRMS-ESI (*m/z*): Calcd. for C_28_H_32_O_7_N_4_F (M+H)^+^: 555.2249; Found 555.2264. M.p.: 68–70 °C.

*7-[3-Amino-4-(2′,3′-dimethoxybenzyloxyimino)piperidin-1-yl]-1-cyclopropyl-6-fluoro-8-methoxy-4-oxo-1,4-dihydroquinoline-3-carboxylic acid* (**19e**). Prepared from **13e** as an off-white solid (18%), m.p.: 73–74 °C. ^1^H-NMR (400 MHz, DMSO-*d_6_*) δ (ppm): 8.70 (1H, s, C_2_-H), 7.78 (1H, d, *J* = 12 Hz, C_5_-H), 6.83 (1H,d, *J* = 8 Hz, Ar-H), 6.74 (1H, t, *J* = 8 Hz, Ar-H ), 6.45 (1H, d, *J* = 8 Hz, Ar-H), 5.11 (2H, s, O-CH_2_Ar), 4.41 (1H, m, cyclopropyl CH), 4.17–4.13 (2H, m, piperidine-H), 3.80 (3H, s, O-CH_3_), 3.74 (6H, s, O-CH_3_), 3.09–3.61 (1H, m, piperidine-H), 2.92–2.81 (1H, m, piperidine-H), 2.75–2.62 (2H, m, piperidine-H), 2.38–2.32 (1H, m, piperidine-H), 1.04–0.96 (4H, m, 2 × cyclopropyl CH_2_). MS-ESI (*m/z*): 555 (M+H)^+^. HRMS-ESI (*m/z*): Calcd. for C_28_H_32_O_7_N_4_F (M+H)^+^: 555.2249; Found 555.2254. M.p.: 73–74 °C.

*7-[3-Amino-4-(3′,4′-dimethoxybenzyloxyimino)piperidin-1-yl]-1-cyclopropyl-6-fluoro-8-methoxy-4-oxo-1,4-dihydroquinoline-3-carboxylic acid* (**19f**). Prepared from **13f** as an off-white solid (20%), m.p.: 88–91 °C. ^1^H-NMR (400 MHz, DMSO-*d_6_*) δ (ppm): 8.69 (1H, s, C_2_-H), 7.83 (1H, d, *J* = 11 Hz, C_5_-H), 6.79 (1H, d, *J* = 1.6 Hz, Ar-H), 6.68 (1H, d, *J* = 8, Ar-H), 6.7 (1H, q, *J_1_* = 8 Hz, *J_2_* = 1.6 Hz, Ar-H), 5.08 (2H, s, O-CH_2_Ar), 4.87 (1H, m, cyclopropyl CH), 4.58 (1H, m, piperidine-H), 4.20–4.16 (1H, m, piperidine-H), 3.77 (9H, O-CH_3_s), 3.53–3.49 (1H, m, piperidine-H), 3.18–3.15 (1H, m, piperidine-H), 3.02–2.96 (1H, m, piperidine-H), 2.82–2.80 (1H, m, piperidine-H), 2.42–2.35 (1H, m, piperidine-H), 1.12–1.04 (4H, m, 2 × cyclopropyl CH_2_). MS-ESI (*m/z*): 555 (M+H)^+^. HRMS-ESI (*m/z*): Calcd. for C_28_H_32_O_7_N_4_F (M+H)^+^: 555.2249; Found 555.2265.

*7-[3-Amino-4-(3′,4′-methylenedioxybenzyloxyimino)piperidin-1-yl]-1-cyclopropyl-6-fluoro-8-methoxy-4-oxo-1,4-dihydroquinoline-3-carboxylic acid* (**19g**). Prepared from **13g** as an off-white solid (25%), m.p.: 79–80 °C. ^1^H-NMR (600 MHz, DMSO-*d_6_*) δ (ppm): 8.68 (1H, s, C_2_-H), 7.78 (1H, d, *J* = 11 Hz, C_5_-H), 6.96 (1H, s, Ar-H), 6.90 (1H, d, *J* = 7.8 Hz, Ar-H), 6.87 (1H, d, *J* = 7.8 Hz, Ar-H), 6.01 (2H, s, OCH_2_O), 4.99 (2H, s, O-CH_2_Ar), 4.83 (1H, m, cyclopropyl CH), 4.17 (1H, m, piperidine-H), 3.74 (3H, s, O-CH_3_), 3.04–3.02 (1H, m, piperidine-H), 2.91–2.88 (1H, m, piperidine-H), 2.82–2.88 (1H, m, piperidine-H), 2.73–2.70 (1H, m, piperidine-H), 2.63–2.60 (1H, m, piperidine-H), 2.38–2.33 (1H, m, piperidine-H), 0.92 (4H, m, 2 × cyclopropyl CH_2_). ^13^C-NMR (150 MHz, DMSO-*d_6_*) δ (ppm): 176.22, 165.60, 157.19 (d, *J* = 268 Hz), 150.57, 150.05, 147.19, 146.92, 146.79, 139.05, 134.09, 133.03, 131.73, 131.55, 121.80, 121.16, 108.71, 106.78 (d, *J* = 24 Hz), 74.75, 63.10, 58.17, 51.31, 49.60, 40.73, 24.39, 9.02. MS-ESI (*m/z*): 539 (M+H)^+^, 561 (M+Na)^+^. HRMS-ESI (*m/z*): Calcd. for C_27_H_28_O_7_N_4_F (M+H)^+^: 539.1941; Found 539.1946.

*7-[3-Amino-4-(2′-methoxybenzyloxyimino)piperidin-1-yl]-1-cyclopropyl-6-fluoro-8-methoxy-4-oxo-1,4-dihydroquinoline-3-carboxylic acid* (**19h**). Prepared from **13h** as an off-white solid (19%), m.p.: 208–211 °C. ^1^H-NMR (400 MHz, DMSO-*d_6_*) δ (ppm): 8.71 (1H, s, C_2_-H), 7.88 (1H, d, *J* = 12 Hz, C_5_-H), 7.34–7.29 (2H, m, Ar-H), 7.03–6.89 (2H, m, Ar-H), 5.12 (2H, s, O-CH_2_Ar), 4.16 (1H, brs, cyclopropyl CH), 3.80–3.68 (5H, m, piperidine-H), 3.50–3.41 (1H, brs, piperidine-H), 3.32 (6H, s, O-CH_3_), 2.05–1.90 (1H, m, piperidine-H), 1.12–0.84 (4H, m, 2 × cyclopropyl CH_2_). MS-ESI (*m/z*): 525 (M+H)^+^, 547 (M+Na)^+^. HRMS-ESI (*m/z*): Calcd. for C_27_H_30_O_6_N_4_F (M+H)^+^: 525.2143; Found 525.2147.

*7-[3-Amino-4-(3′-methoxybenzyloxyimino)piperidin-1-yl]-1-cyclopropyl-6-fluoro-8-methoxy-4-oxo-1,4-dihydroquinoline-3-carboxylic acid* (**19i**). Prepared from **13i** as an off-white solid (23%), m.p.: 94–95 °C. ^1^H-NMR (600 MHz, DMSO-*d_6_*) δ (ppm): 8.65 (1H, s, C_2_-H), 7.75 (1H, d, *J* = 11 Hz, C_5_-H), 7.14 (1H, s, Ar-H), 6.86–6.83 (3H, m, Ar-H), 5.03 (2H, s, O-CH_2_Ar), 4.43–4.41 (1H, brs, cyclopropyl CH), 4.17–4.14 (1H, brs, piperidine-H), 3.75 (6H, s, O-CH_3_), 3.45–3.43 (1H, m, piperidine-H), 3.23–3.18 (1H, m, piperidine-H), 3.06–3.04 (1H, m, piperidine-H), 2.87–2.84 (1H, m, piperidine-H), 2.70–2.66 (1H, m, piperidine-H), 2.34–2.29 (1H, m, piperidine-H), 1.17–1.12 (4H, m, 2 × cyclopropyl CH_2_). ^13^C-NMR (150 MHz, DMSO-*d6*) δ (ppm): 176.23, 165.83, 165.60, 158.29 (d, *J* = 235 Hz), 156.98, 154.63, 150.63, 150.12, 129.88, 129.76, 129.24, 113.68, 113.29, 106.92, 106.76, 106.63, 106.25 (d, *J* = 25 Hz), 74.36, 63.18, 55.06, 54.87, 49.57, 45.27, 40.73, 24.50, 8.59. MS-ESI (*m/z*): 525 (M+H)^+^, 547 (M+Na)^+^. HRMS-ESI (*m/z*): Calcd. for C_27_H_30_O_6_N_4_F (M+H)^+^: 525.2143; Found 525.2149.

*7-[3-Amino-4-(4′-methoxybenzyloxyimino)piperidin-1-yl]-1-cyclopropyl-6-fluoro-8-methoxy-4-oxo-1,4-dihydroquinoline-3-carboxylic acid* (**19j**). Prepared from **13j** as an off-white solid (27%), m.p.: 80–82 °C. ^1^H-NMR (400 MHz, DMSO-*d_6_*) δ (ppm): 8.66 (1H, s, C_2_-H), 7.76–7.68 (1H, m, C_5_-H), 7.31 (2H, d, *J* = 8 Hz, Ar-H), 6.91 (2H, d, *J* = 8 Hz, Ar-H), 4.99 (2H, s, O-CH_2_Ar), 4.14 (1H, brs, cyclopropyl CH), 3.74 (6H, s, O-CH_3_), 3.65 (1H, m, piperidine-H), 3.37 (1H, brs, piperidine-H), 3.27 (1H, brs, piperidine-H), 3.16 (1H, brs, piperidine-H), 2.99 (1H, brs, piperidine-H), 2.30 (1H, m, piperidine-H), 1.96 (1H, m, piperidine-H), 1.09–1.02 (4H, m, 2 × cyclopropyl CH_2_). ^13^C-NMR (150 MHz, DMSO-*d_6_*) δ (ppm): 176.32, 165.71, 158.91, 153.05 (d, *J* = 250 Hz), 150.58, 134.09, 129.82, 129.65, 129.23, 122.12, 113.65, 107.00, 106.48 (d, *J* = 22 Hz), 74.63, 63.08, 55.04, 51.44, 49.64, 45.40, 25.09, 8.90. MS-ESI (*m/z*): 525 (M+H)^+^. HRMS-ESI (*m/z*): Calcd. for C_27_H_30_O_6_N_4_F (M+H)^+^: 525.2143; Found 525.2146.

*7-[3-Amino-4-(4′-flourobenzyloxyimino)piperidin-1-yl]-1-cyclopropyl-6-fluoro-8-methoxy-4-oxo-1,4-dihydroquinoline-3-carboxylic acid* (**19k**). Prepared from **13k** as an off-white solid (30%), m.p.: 74–77 °C. ^1^H-NMR (400 MHz, DMSO-*d_6_*) δ (ppm): 8.63 (1H, s, C_2_-H), 7.77 (1H, d, *J* =12 Hz, C_5_-H), 7.45 (2H, m, Ar-H), 7.11 (2H, m, Ar-H), 5.09 (2H, s, O-CH_2_Ar), 4.91(1H, m, cyclopropyl CH), 4.11 (1H, m, piperidine-H), 3.73 (3H, s, O-CH_3_), 3.47 (1H, m, piperidine-H), 3.25 (1H, m, piperidine-H), 3.12 (1H, m, piperidine-H), 2.91 (1H, m, piperidine-H), 2.72 (1H, m, piperidine-H), 2.35 (1H, m, piperidine-H), 0.96 (4H, m, 2 × cyclopropyl CH_2_). ^13^C-NMR (150 MHz, DMSO-*d_6_*) δ (ppm): 176.32, 165.56, 163.78 (d, *J* = 243 Hz), 155.42 (d, *J* = 247 Hz), 150.68, 149.61, 145.21, 133.75, 133.04, 130.24, 129.62, 129.54, 115.22, 114.62, 106.31 (d, *J* = 24 Hz), 74.43, 63.25, 55.98, 50.23, 49.52, 40.94, 24.71, 8.67. MS-ESI (*m/z*): 513 (M+H)^+^, 535 (M+Na)^+^. HRMS-ESI (*m/z*): Calcd. for C_26_H_27_O_5_N_4_F_2_ (M+H)^+^: 513.1949; Found 513.1952.

*7-[3-Amino-4-(4′-chlorobenzyloxyimino)piperidin-1-yl]-1-cyclopropyl-6-fluoro-8-methoxy-4-oxo-1,4-dihydroquinoline-3-carboxylic acid* (**19l**). Prepared from **13l** as an off-white solid (34%), m.p.: 67–69 °C. ^1^H-NMR (400 MHz, DMSO-*d_6_*) δ (ppm): 8.67 (1H, s, C_2_-H), 7.79 (1H, d, *J* = 12 Hz, C_5_-H), 7.14 (2H, m, Ar-H), 7.07 (2H, m, Ar-H), 5.11 (2H, s, O-CH_2_Ar), 4.94 (1H, m, cyclopropyl CH), 4.40 (1H, m, piperidine-H), 4.01 (1H, m, piperidine-H), 3.75 (3H, s, O-CH_3_), 3.18–3.14 (1H, m, piperidine-H), 3.07–3.05 (1H, m, piperidine-H), 2.92–2.90 (1H, m, piperidine-H), 2.79–2.76 (1H, m, piperidine-H), 2.43–2.38 (1H, m, piperidine-H), 1.07–1.04 (4H, m, 2 × cyclopropyl CH_2_). ^13^C-NMR (150 MHz, DMSO-*d_6_*) δ (ppm): 176.18, 165.59, 157.75, 155.47 (d, *J* = 248 Hz), 150.59, 146.11, 137.12, 134.08, 132.25, 129.79, 129.00, 121.19, 117.10, 106.67 (d, *J* = 23 Hz), 106.61, 73.98, 63.13, 57.79, 51.14, 45.26, 40.72, 24.91, 8.86. MS-ESI (*m/z*): 529 (M+H)^+^. HRMS-ESI (*m/z*): Calcd. for C_26_H_27_O_5_N_4_FCl (M+H)^+^: 529.1648; Found 529.1637.

*7-(3-Amino-4-benzyloxyiminopiperidin-1-yl)-1-cyclopropyl-6-fluoro-8-methoxy-4-oxo-1,4-dihydro-quinoline-3-carboxylic acid* (**19m**). Prepared from **13m** as an off-white solid (32%), m.p.: 84–85 °C. ^1^H-NMR (600 MHz, DMSO-*d_6_*) δ (ppm): 8.68 (1H, s, C_2_-H), 8.74 (1H, d, *J* = 12 Hz, C_5_-H), 7.29–7.21 (5H, m, Ar-H), 5.10 (2H, s, O-CH_2_Ar), 4.97–4.92 (1H, m, cyclopropyl CH), 4.43–4.39 (1H, m, piperidine-H), 4.16–4.13 (1H, m, piperidine-H), 3.72 (3H, s, O-CH_3_), 3.65–3.63 (1H, m, piperidine-H), 3.2–3.15 (1H, m, piperidine-H), 2.86–2.83 (1H, m, piperidine-H), 2.71–2.67 (1H, m, piperidine-H), 2.37–2.34 (1H, m, piperidine-H), 1.08–0.95 (4H, m, 2 × cyclopropyl CH_2_). ^13^C-NMR (150 MHz, DMSO-*d6*) δ (ppm): 176.26, 165.73, 155.65 (d, *J* = 255 Hz), 150.60, 150.19, 146.05, 137.89, 133.09, 128.14, 127.98, 127.68, 127.73, 127.29, 106.86 (d, *J* = 21 Hz), 106.35, 74.62, 61.29, 53.11, 51.35, 45.39, 40.75, 24.78, 8.98. MS-ESI (*m/z*): 495 (M+H)^+^, 517 (M+Na)^+^. HRMS-ESI (*m/z*): Calcd. for C_26_H_28_O_5_N_4_F (M+H)^+^: 495.2038; Found 495.2048. M.p.: 84–85 °C.

### 3.3. MIC Determination

Compounds **19a**–**m** were evaluated for their *in vitro* antibacterial activity using standard techniques in comparison to the reference drugs IMB-070593, CPFX and LVFX. Drugs (10.0 mg) were dissolved in 0.1 N NaOH solution and water (10 mL). Further progressive two fold serial dilution with melted Mueller-Hinton agar was performed to obtain the required concentrations of 128, 64, 32, 16, 8, 4, 2, 1, 0.5, 0.25, 0.125, 0.06, 0.03, 0.015 and 0.008 mg/mL. Petri dishes were incubated with 104 colony-forming units (cfu) and incubated at 35 °C for 18 h. MIC was the lowest concentration of the test compound, which resulted in no visible growth on the plate.

## 4. Conclusions

In summary, a series of novel IMB-070593 derivatives with remarkable improvement in lipophilicity, as compared to the parent IMB-070593, were designed, synthesized and evaluated for their *in vitro* anti-MTB and antibacterial activity. Our results reveal that compound **19l** has good *in vitro* activity against MTB H37Rv ATCC 27294 (MIC: 0.125 µg/mL) which is 2–4 fold more potent than the parent IMB070593, CPFX and LVFX (MICs: 0.25–0.5 µg/mL). Whereas, compounds **19h**, **19j**, **19k** and **19m** show interesting antibacterial activity (MICs: <0.008–4 µg/mL) against all of the tested Gram-positive strains including CPFX- and/or LVFX-resistant MSSA, MRSA and MSSE. However, the target compounds **19a**–**m** are generally less active than IMB-070593 against MDR-MTB 20161 and Gram-negative strains. It suggests that merely an increase in lipophilicity of the tested compounds does not result in enhanced antimycobacterial and antibacterial activities.

## References

[1] Lv K., Sun Y.X., Sun L.Y., Guo H.Y., Wu J.W., Liu M.L. (2012). Design, synthesis and *in vitro* antibacterial activity of fluoroquinolone derivatives containing a chiral 3-(alkoxyimino)-2-(aminomethyl)azetidine moiety. ChemMedChem.

[2] Aubry A., Pan X.S., Aubry L.M., Jarlier V., Emmanuelle C. (2004). Mycobacterium tuberculosis DNA Gyrase: Interaction with quinolones and correlation with antimycobacterial drug activity. Antimicrob. Agents Chemother..

[3] Crofton J., Choculet P., Maher D. (1997). Guidelines for the Management of Drug-Resistant Tuberculosis.

[4] Bradbury B.J., Pucci M.J. (2008). Recent advances in bacterial topoisomerase inhibitors. Curr. Opin. Pharmacol..

[5] Ginsburg A.S., Grosset J.H., Bishai W.R. (2003). Fluoroquinolones, tuberculosis, and resistance. Lancet Infect. Dis..

[6] Dalhoff A. (2012). Resistance surveillance studies: A multifaceted problem—The fluoroquinolone example. Infection.

[7] Grimaldo E.R., Tupasi T.E., Rivera A.B., Quelapio M.I. D., Cardano R.C., Derilo J.O., BelenIncreased V.A. (2001). Increased resistance to ciprofloxacin and ofloxacin in multidrug-resistant *Mycobacterium tuberculosis* isolates from patients seen at a tertiary hospital in the Philippines. Int. J. Tuberc. Lung. Dis..

[8] Lv K., Wu J.W., Wang J., Liu M.L., Wei Z.Q., Cao J., Sun Y.X., Guo H.Y. (2013). Synthesis and *in vitro* antibacterial activity of quinolone/naphthyridone derivatives containing 3-alkoxyimino-4-(methyl)aminopiperidine scaffolds. Bioorg. Med. Chem. Lett..

[9] Zhang Y.B., Liu M.L., Guo H.Y. (2008). Structure characteristics of quinolones and structure-activity relationships against Gram-positive bacteria. World Notes Antibiot..

[10] Liu M.L., Guo H.Y. (2006). Non-fluoroquinolones: discovery and structure-activity relationship studies. World Notes Antibiot..

[11] Chai Y., Liu M.L., Lv K., Feng L.S., Li S.J., Sun L.Y., Wang S., Guo H.Y. (2011). Synthesis and *in vitro* antibacterial activity of a series of novel gatifloxacin derivatives. Eur. J. Med. Chem..

[12] Feng L.S., Liu M.L., Wang S., Chai Y., Li S.J., Guo H.Y. (2012). Synthesis and *in vitro* antimycobacterial activity of moxifloxacin methylene and ethylene isatin derivatives. Chem. Res. Chin. Univ..

[13] Sharma P.C., Jain A., Jain S., Pahwa R., Yar M.S. (2010). Ciprofloxacin: Review on developments in synthetic, analytical, and medicinal aspects. J. Enzyme Inhib. Med. Chem..

[14] Sriram D., Yogeeswari P., Basha J.S., Radha D.R., Nagaraja V.J. (2005). Synthesis and antimycobacterial evaluation of various 7-substituted ciprofloxacin derivatives. Bioorg. Med. Chem..

[15] Sriram D., Aubry A., Yogeeswaria P., Fisher L.M. (2006). Gatifloxacin derivatives: Synthesis, antimycobacterial activities, and inhibition of Mycobacterium tuberculosis DNA gyras. Bioorg. Med. Chem. Lett..

[16] Feng L.-S., Liu M.-L., Zhang S., Chai Y., Wang B., Zhang Y.-B., Lv K., Guan Y., Guo H.-Y., Xiao C.-L. (2011). Synthesis and *in vitro* antimycobacterial activity of 8-OCH_3_ ciprofloxacin methylene and ethylene isatin derivatives. Eur. J. Med. Chem..

[17] Guo Q., Liu M.-L., Feng L.-S., Lv K., Guan Y., Guo H.-Y., Xiao C.-L. (2011). Synthesis and *in-vitro* antimycobacterial activity of fluoroquinolone derivatives containing a coumarin moiety. Arch. Pharm..

[18] Feng L.S., Liu M.L., Wang B., Chai Y., Hao X.Q., Meng S., Guo H.Y. (2010). Synthesis and *in vitro* antimycobacterial activity of balofloxacin ethylene isatin derivatives. Eur. J. Med. Chem..

[19] Feng L.S., Lv K., Liu M.L., Wang S., Zhao J., You X.F., Li S.J., Cao J., Guo H.Y. (2012). Synthesis and *in vitro* antibacterial activity of gemifloxacin derivatives containing a substituted benzyloxime moiety. Eur. J. Med. Chem..

[20] Wang X.Y., Guo Q., Wang Y.C., Liu B.Q., Liu M.L., Sun L.Y., Guo H.Y. (2008). Synthesis and antibacterial activity of 7-(4-alkoxyimino-3-aminopiperidin-1-yl)fluoroquinolone derivatives. Acta Pharm. Sin..

[21] Gui H.Y., Liu M.L., Liu B.Q., Hu J.S., Wu J.W., Wang Z. (2010). Application of 7-(4-alkoxyimino-3-aminopiperidin-1-yl)fluoroquinolones and their combinations. CN Patent.

[22] Brown P., Calvert S.H., Chapman P.C.A., Cosham S.C., Eglington A.J., Elliot R.L., Harris M.A., Hinks J.D., Lowther J., Merrikin D.J. (1991). β-Lactamase-stable penicillins. Synthesis and structure-activity relationships of (*Z*)-alkyloxyimino penicillins; selection of BRL 44154. J. Chem. Soc. Perkin Trans. 1.

[23] Chai Y., Liu M.L., Wang B., You X.F., Feng L.S., Zhang Y.B., Cao J., Guo H.Y. (2010). Synthesis and *in vitro* antibacterial activity of novel fluoroquinolone derivatives containing substituted piperidines. Bioorg. Med. Chem. Lett..

[24] Chai Y., Wan Z.L., Wang B., Guo H.Y., Liu M.L. (2009). Synthesis and *in vitro* antibacterial activity of 7-(4-alkoxyimino-3-amino-3-methylpiperidin-1-yl)fluoroquinolone derivatives. Eur. J. Med. Chem..

[25] Collins L., Franzblau S.G. (1997). Microplate alamar blue assay *versus* BACTEC 460 system for high-throughput screening of compounds against *Mycobacterium tuberculosis* and *Mycobacterium avium*. Antimicrob. Agents Chemother..

[26] Lu Y., Zheng M.Q., Wang B., Fu L., Zhao W.J., Li P., Xu J., Zhu H., Jin H.X., Yin D.L. (2011). Clofazimine analogs with efficacy against experimental tuberculosis and reduced potential for accumulation. Antimicrob. Agents Chemother..

[27] (2001). National Committee for Clinical Laboratory Standards, Performance Standards for Antimicrobial Susceptibility Testing: 11th Informational Supplement, Volume 21.

